# To halt disease progression rehabilitation in MS should start early: No

**DOI:** 10.1177/13524585241289277

**Published:** 2024-11-15

**Authors:** Declan T Chard

**Affiliations:** NMR Research Unit, Queen Square MS Centre, Department of Neuroinflammation, UCL Queen Square Institute of Neurology, Faculty of Brain Sciences, University College London, London, UK; National Institute for Health Research (NIHR) University College London Hospitals (UCLH) Biomedical Research Centre, London, UK

In multiple sclerosis (MS) neurodegeneration is thought to be responsible for the majority of progressive irreversible disability.^1^ The main cause of neurodegeneration in MS is unknown, and it is likely to be multifactorial: MS lesion formation is associated with axonal transection, and ongoing axonal loss is observed in those that remyelinate incompletely or become chronically active;^2^ beyond lesions there is widespread neuro-axonal loss that cannot readily be explained by the effects of lesions alone, and other factors appear to be at work.^2^ To stop progression, it is likely that multiple ongoing pathological processes will have to be addressed and, where significant tissue damage has already been accrued, repair promoted. As such, it seems doubtful that any treatment, be this pharmacological or not, could be expected to arrest progression entirely on its own, and that different combinations of treatments may be relevant dependent on the main pathological processes underlying progression in each person with MS at any given time.

How early should we start treatment to halt progression? Several lines of evidence suggest that there is scope for interventions to be relevant soon after symptoms first occur, and not just in people with clinically evident progression. In people with relapsing-remitting MS, in addition to substantial progression independent of relapses,^3^ accelerated brain atrophy (the main magnetic resonance imaging marker of neurodegeneration) is detectable soon after first symptom onset, even though it may be more than a decade before progressive disability dominates clinically (e.g. Haider et al.^4^). There is also the emerging concept of ‘network collapse’, the point beyond which neural networks can no longer compensate for further damage,^5^ and so the opportunity for a treatment to have its greatest beneficial effect before this occurs. Taken together, it can be argued that preventive management should be considered for those most at risk of progression even before it occurs, rather than aiming to act against it once established. In this context, there would be nothing to rehabilitate, and instead the goal of a non-pharmacological intervention would be to improve functional resilience to pathology. Here, it is worth noting the concept of brain reserve, the capacity of a person’s brain to compensate for, and adapt to, pathology before it starts to have clinically detectable effects: Reducing cardiovascular risk factors, and improving diet, exercise and sleep, may all help to preserve this.^6^

Does rehabilitation have a disease-modifying effect in MS? While it does have detectable effects on brain networks and is associated with improved function (e.g. Tavazzi et al.^7^), to the best of my knowledge, it has not been shown that this translates into a sustained impact on the subsequent disease course. There is some evidence that exercise, rather than rehabilitation per se, may influence the innate immune system in MS,^8^ but this does not appear to significantly reduce the accrual of white matter brain lesions or brain atrophy.^9^ Further work is needed to look for a disease-modifying effect of rehabilitation (or exercise) and, importantly, determine if this is of a sufficient magnitude to be clinically meaningful on its own or in combination with medications. However, controlled trials are challenging as placebo interventions may themselves have therapeutic effects.^10^

In conclusion, while rehabilitation has an established and valuable role restoring and preserving function in MS, and on this basis needs no other justification in clinical practice, at present there is no definitive evidence that it has a persistent disease-modifying effect, and by the time functional deficits are apparent (and so rehabilitation indicated), the optimal window of opportunity to halt progression may already have passed. There are also practical considerations: Rehabilitation programmes are time and energy consuming, and for people with MS balancing many significant demands for both with sometimes substantial fatigue, unless rehabilitation has compelling benefits (e.g. promoting functional recovery following a relapse) compared with other treatment options then it may not be a priority.
